# LiPheStream - A 18-month high spatiotemporal resolution point cloud time series of Boreal trees from Finland

**DOI:** 10.1038/s41597-024-04143-w

**Published:** 2024-11-25

**Authors:** Samantha Wittke, Mariana Campos, Lassi Ruoppa, Rami Echriti, Yunsheng Wang, Antoni Gołoś, Antero Kukko, Juha Hyyppä, Eetu Puttonen

**Affiliations:** 1 Finnish Geospatial Research Institute in the National Land Survey of Finland, Department of Remote Sensing and Photogrammetry, Espoo, 02150 Finland; 2 Aalto University, Department of Built Environment, Espoo, 02150 Finland; 3 CSC-IT Center for Science Ltd., 02101 Espoo, Finland; 4 Aalto University, Department of Mathematics and Systems Analysis, Espoo, 02150 Finland

**Keywords:** Forest ecology, Forestry

## Abstract

In the present paper, we introduce a high-resolution spatiotemporal point cloud time series, acquired using a LiDAR sensor mounted 30 metres above ground on a flux observation tower monitoring a boreal forest. The dataset comprises a 18-month long (April 2020 - September 2021) time series with an average interval of 3.5 days between observations. The data acquisition, transfer, and storage systems established at Hyytiälä (Finland) are named the LiDAR Phenology station (LiPhe). The dataset consists of 103 time points of LiDAR point clouds covering a total of 458 individual trees, comprising three distinct Boreal species. Additional reference information includes the respective location, the species, and the initial height (at the first time point) of each individual tree. The processing scripts are included to outline the workflow used to generate the individual tree point clouds (LiPheKit). The presented dataset offers a comprehensive insight into inter- and intra-species variations of the individual trees regarding their growth strategies, phenological dynamics, and other functioning processes over two growth seasons.

## Background & Summary

Climate change affects forest ecosystems and tree functioning processes. For example, forests under climatic stress may become more susceptible to reduced resilience^1^, biodiversity loss^2^, and variations in phenological cycles^3^. Therefore, timely identification and understanding of new stressors and their impacts on forests need continuous large-scale monitoring. Recent efforts have been made to develop standardized variables for forest monitoring, with a particular emphasis on comprehending the impacts of climate, biodiversity, and other environmental factors on global scale, including essential biodiversity variables (EBV)^4^. Nowadays, EBV, such as ecosystem disturbances, phenology and vertical profile, are often monitored by Earth observation (EO) platforms with passive sensors^5^, especially at global coverage. However, many uncertainties exist about the accuracy of estimating these EBV, leading to substantial doubts in the projections of future climate change effects on forests^6^. In fact, many forest events and dynamics cannot be directly detected at the temporal and spatial resolutions of Earth observation. First, the temporal resolution depends on the satellite revisit time, which can be longer than many events, such as phenological changes, and on the meteorological conditions (e.g. daytime and clouds) that can decrease the amount of useful data. Second, land surface species heterogeneity is undetectable at satellite images spatial resolution. The spectral response of different tree species is mixed in the same pixel, which hampers the understanding of individual species responses to extreme events and species-specific analyses. Therefore, in general, the essential variable estimates based on satellite images require a comparison with independent data to support forest dynamics modelling and analyses. The lack of adequate ground truth forest data has been highlighted as the major bottleneck to interpreting and validating climate change effects on forests estimated based on satellite-derived time series^7,8^.

Close-range sensing techniques that observe the targets from a distance of less than a few kilometres using active or passive sensors provide a bridge between ground-based observations and satellite-based imagery. Previous works prove the capability of close-range remote sensors to detect forest dynamics and estimate EBV^6,9–11^. Close-range data acquisitions have significantly higher spatial resolution and dimension than satellite remote sensing metrics. Additionally, the automation of remote control of close-range sensors and the capacity to handle large amounts of data have significantly advanced. Thus, continuous monitoring of forest dynamics by near-ground remote sensors and over an extended period of time is nowadays possible. However, the potential of close-range sensing in providing a long-term time series with high spatial (centimetre-level) and temporal (hour-level) resolution observation to accurately detect and estimate forest dynamics remains largely unexplored^12^. The geographical and temporal coverage, as well as the data management and sensor maintenance costs, remain as limitations of close-range networks. For example, the largest close-range sensing network, Phenocam, also relies on passive sensors^6,13–15^. Adverse lighting conditions lead to an increase in random gaps in their time series. Additionally, the single-camera setup only offers radiometric information of 2D images (such as pixel colours and vegetation indices), while geometric information, such as three-dimensional structural information, remains unavailable.

Light Detection and Ranging (LiDAR) is an active scanning technique and complements the earlier optical techniques with its capability of providing 3D structural information of objects in adverse lighting conditions. More specifically for mapping forest ecosystem structure, LiDAR is particularly effective at estimating volume, plant and leaf area indexes and biomass both globally with satellites and locally from airborne or ground platforms^16–19^. These parameters are derived from the 3D geometrical information. While the usability of long-term LiDAR time series is recognized, instrumentation dedicated to systematic multi-year forest monitoring is limited. These limitations derive from the LiDAR data collection costs, large data volumes, and the high data processing requirements due to target complexity leading to trade-offs in experiment design. The majority of studies circumvent these challenges by limiting either the temporal or spatial resolution of LiDAR time series. Forest LiDAR campaigns with individual tree resolution are typically conducted annually or, at best, with two time points per year. Although this limited temporal resolution is usually adequate for capturing overall forest growth and estimating changes in above-ground biomass (AGB), it may miss ecophysiological processes occurring throughout the year. Alternatively, previous studies suggested establishing a network of low-spatial resolution independent scanners^11,20–22^ that offer dense temporal coverage and adequate spatial resolution for monitoring plant and leaf area densities and indexes around the scanner, but finer changes in tree canopy structure are not noticeable.

More recently, case studies employing permanent laser scanning (PLS) for natural process monitoring, such as beachlines, landslides, and glacier dynamics^23–28^, have demonstrated the utility of dense spatiotemporal point cloud time series in extended high precision monitoring. In the same direction, the Finnish Geospatial Research Institute (FGI) Lidar Phenology station (LiPhe) is a PLS platform developed to collect dense spatiotemporal time series from a Boreal forest^29^. The LiPhe platform was developed based on the experiences from individual terrestrial laser scanning (TLS) studies that have reported and quantified individual branch movements over time in different natural and laboratory settings^30–35^. Single tree datasets often capture the trees once or maybe twice in their lifetime^11,36–40^ and are well suited for studies focusing on individual tree or forest structure, or determining their current state. These datasets however, do not enable observation of tree phenology over a growth season or what their response mechanisms are in a changing environment. Studies utilizing data from the LiPhe station have shown the potential of a PLS system capable of providing a multitemporal single tree dataset in understanding the annual structural dynamics in a forest^41–43^.

The present paper introduces the “LiPheStream” dataset^44^, a 18-month-long point cloud time series comprising LiDAR point cloud data at 103 time points for 458 individual trees that consisted of three Boreal species (silver birch (*Betula pendula* Roth.), Scots pine (*Pinus sylvestris* L.) and Norway spruce (*Picea abies H.* Karst.)). The dataset was collected by the LiPhe station between April 2020 and September 2021. In addition, we provide a set of tools (named LiPheKit) used for producing the dataset including practical examples on how to query the datasets. Previous works had especially focused on terrestrial^45^, UAV^46^ or multi-platform laser scanning^37,47^ individual tree point clouds datasets. This dataset initiative aims to contribute to the advancement of our understanding as ecologist and remote sensing communities of PLS time-series potential for investigating how forests adapt to climatic stress factors. The LiPheStream^44^ provides an opportunity to enhance existing point cloud processing software for registering complex natural scenes and generating simulated point clouds, for instance, to build more accurate digital forest twins. Moreover, the LiPheStream can provide valuable insights for designing improved monitoring experiments that optimize timing and spatio-temporal resolution necessary to capture forest structure and changes relevant to EBV.

## Methods

In this section, the design, data acquisition setup and data processing pipeline performed to generate the LiPheStream dataset^44^ from the LiPhe PLS is described in detail. The tree level dataset provided in this paper is a curated subset of the 2020-2021 data collection period. More details about data curation are presented in the Data Selection section.

### LiPhe data acquisition setup

The FGI LiDAR phenology station (LiPhe)^29^ is a PLS system established in 2020 at the Hyytiälä forest research station (University of Helsinki) located in southern Finland. The aim of the LiPhe station is to monitor daily and seasonal vegetation dynamics through continuous monitoring of a fixed forest scene. LiPhe is the first PLS system designed for forest applications and the only one in operation at the present moment. LiPhe hardware includes a time-of-flight laser scanner (RIEGL VZ-2000i scanner, RIEGL Laser Measurement Systems GmbH, Austria), weather-protected hood, 24V power supply, local measurement computer (local server) and a permanent network storage (NAS). The laser scanner with the weather-protected hood was permanently mounted at 30 metres from ground in a 35-metre observation tower^29^. The scanner was tilted 60 degrees down to monitor the forest with a unique above and oblique forest canopy perspective.

Aiming at long-term forest monitoring with centimetre positional and hour-level temporal resolution, every 60 minutes the forest scene is scanned with a 0.006° angular resolution and a 1,200 kHz scan frequency. RIEGL VZ-2000i is a long-range scanner, which enables a measurement range of up to 2,500 meters. The nominal 3D position accuracy is 5 mm at 100 meters with a beam divergence ranges from 0.19 to 0.27 mrad. With an angular resolution of 0.006 degrees, the 3D point spacing is smaller than 1 cm at 100 meters. The clearer the line of sight between a tree and the scanner, the higher the quality level of the tree (spatial resolution and completeness). However, the single-view perspective with only one side facing the scanner and inherent occlusions can affect the completeness of the tree data, which is an important limitation of the LiPheStream dataset and needs to be taken into account in different use cases.

The repeated high-resolution data collection setup produces large datasets whose transferring, storage and data processing also present a challenge. LiPhe scanning and data transfer are fully automated by Python scripts. After each scan acquisition, the scan data and metadata are transferred to the local measurement computer located at the tower (10GB per scan), while the transfer to permanent network storage happens once per day (full project with 24 scans - 240 GB per day). Thus, the LiPhe scan data used in the presented dataset is a subset collected between April 2020 and September 2021. More time points are available upon request. More technical details about LiPhe hardware components and data acquisition setup can be found at^29^.

**Fig. 1:**
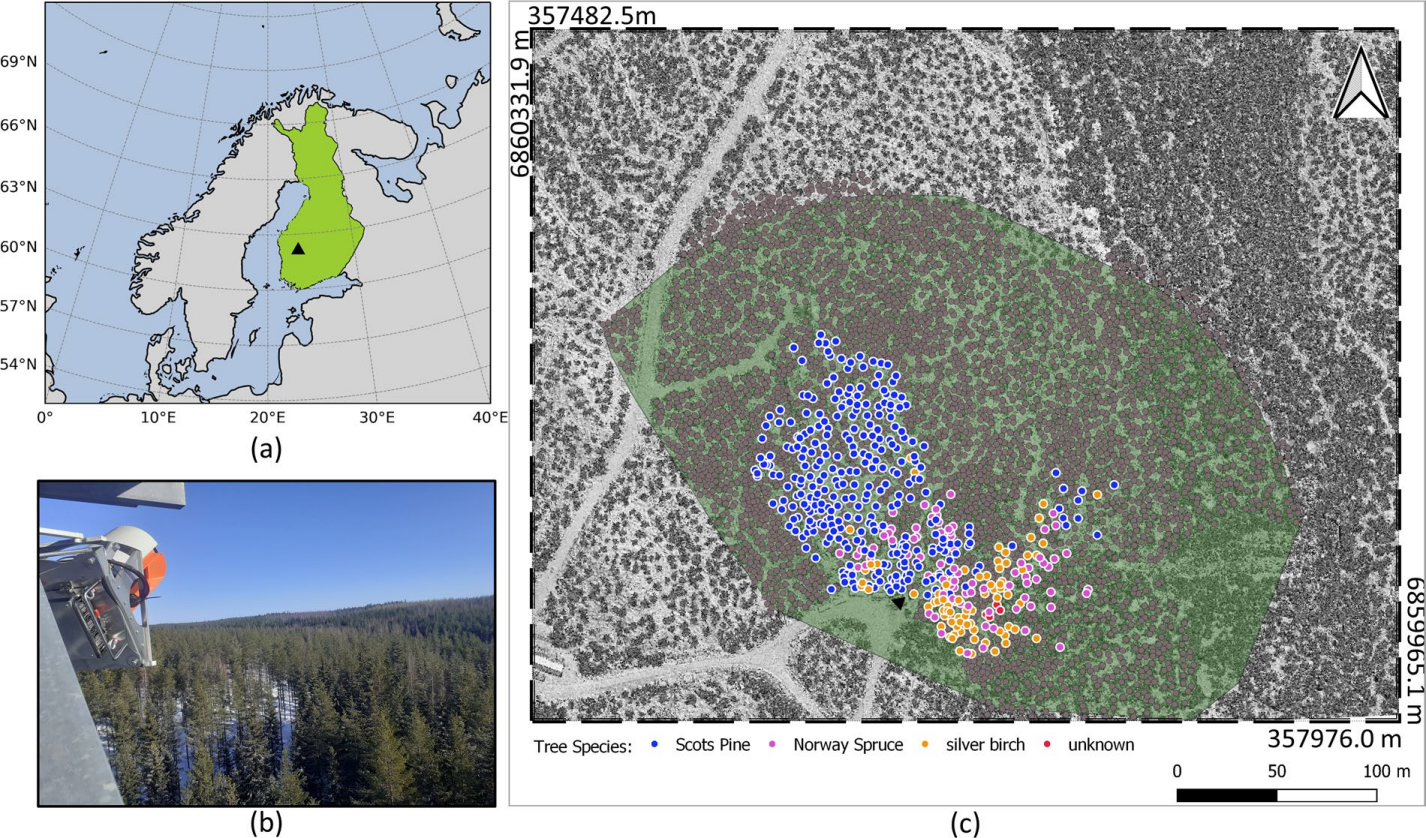
LiPheStream dataset on the map: (**a**) Approximate location of Hyytiälä forest research station (marked by a black triangle) within Finland (highlighted in green). (**b**) LiPhe station scanner observing the monitored trees. (**c**) Selected trees available in LiPheStream, colorized by species, within the scanned area at Hyytiälä forest research station (marked by a green buffer) and LiPhe station location (black triangle). The three tree species, Scots pine, Norway spruce, and silver birch, are represented by blue, magenta and yellow dots, respectively.

Figure 1 shows in panel (a) the Hyytiälä forest station (https://www.helsinki.fi/en/research-stations/hyytiala-forest-station) (61°51′ N, 24°17′ E) location on the map of Finland represented by a black triangle. Panel (b) shows the RIEGL VZ-2000i scanner installed on a 35-meter observation tower, above the tree canopy and with field of view oriented towards the forest. Panel (c) displays the LiPhe coverage area within a 200 m radius of the laser scanner system (shown in transparent green) superimposed on a digital surface model (DSM) of the research area. LiPhe position is represented by the black triangle, while, the tree species are represented by blue, magenta and yellow dots, corresponding to Scots pine, Norway spruce, and silver birch, respectively.

### LiPhe data processing: from full scene to individual tree point clouds

**Fig. 2:**
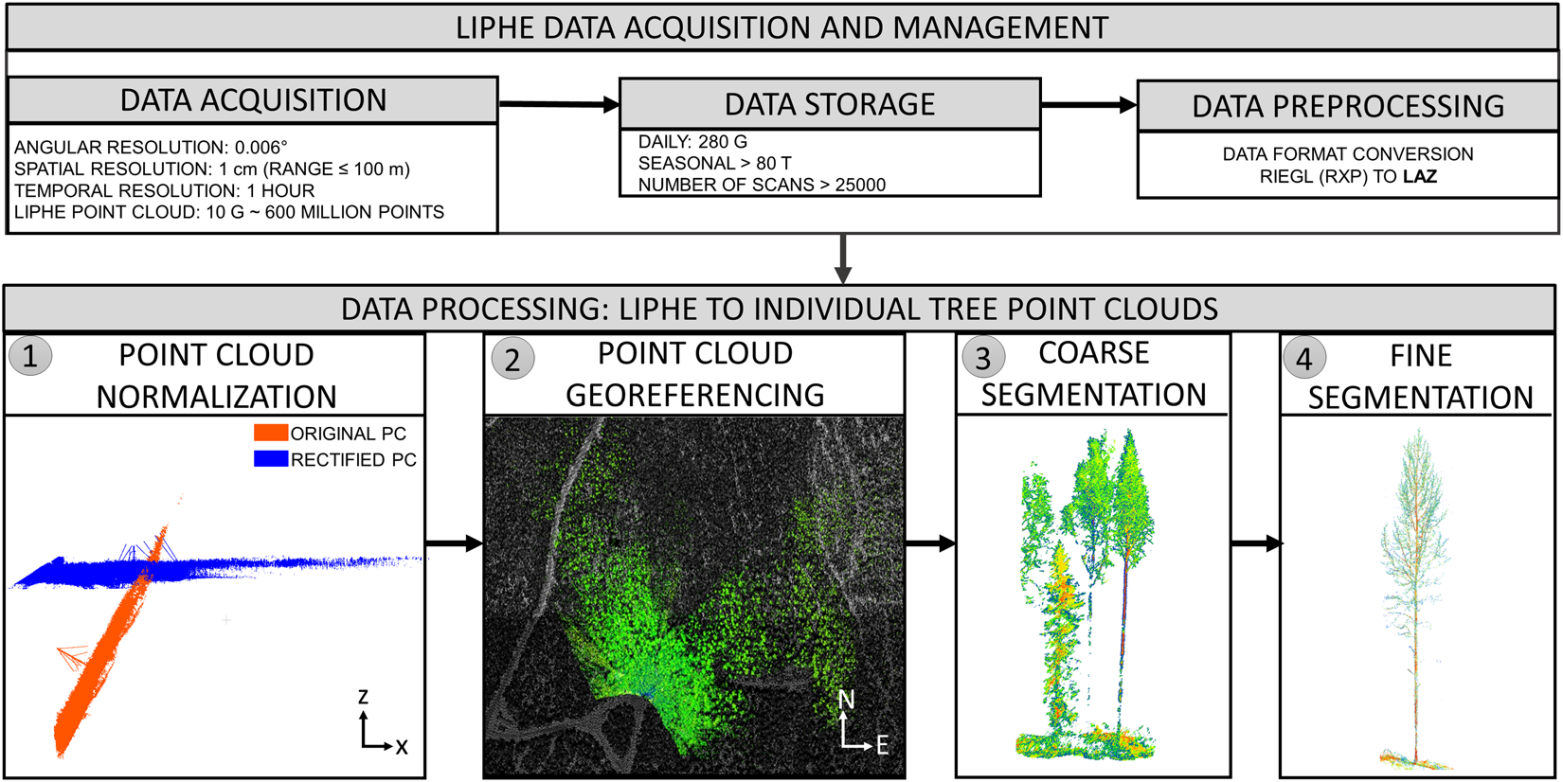
Example schema from LiPhe data acquisition to the LiPheStream dataset. The data processing workflow show the sequential steps: point cloud normalization, georeferencing, coarse segmentation, and fine segmentation.

The current data processing pipeline includes data conversion from RIEGL’s proprietary data format (*rxp*) to the compressed ASPRS *las* format (*laz*), point cloud normalization, registration and georeferencing, data resampling, coarse-to-fine point cloud segmentation and tree parameter estimation (e.g. tree height), which is summarized in Fig. 2. The pipeline, named “LiPheKit”, was developed in Python programming language. The pipeline code and usage information can be found at the Code availability section. The data processing will be described, starting from the LiPhe point cloud already converted to *laz* format. The preprocessing step where the raw scanner data (RIEGL *rxp* format) is converted into *laz* format is omitted. The *rxp*-to-*laz* conversion using combined C++ and Python functions requires proprietary RIEGL libraries, RDBLib and RiMTA, which are not openly available.

Since the scan hood is tilted 60 degrees down and located 30 m from the ground, the original point clouds are tilted in relation to the ground plane with the scanner position placed in the origin. Therefore, as a first step, the point clouds were rectified for a local reference system normalized to the ground. A 3D passive rotation was performed to normalize the full point cloud to the ground, in which a right-hand system was defined with origin at the scan and Z to up. The origin at the scan is located 30 m from the ground (tower), providing negative Z values. The rotation parameters (euler angles) were Omega = 0 degrees; phi = 60 degrees and kappa = 90 degrees. During the rectification process, extra parameters such as scan angles and range are also computed from the 3D coordinates (X, Y, Z) and added to the point clouds. While the point amplitude values were saved as intensity, scan angles (theta and phi in degrees), reflectance (dB), return pulse deviation (measure of pulse shape distortion) and range (m) were saved as extra parameters in the *laz* file. LiDAR intensity has been defined as the reflected energy of the emitted laser beam^48^. LiDAR calibrated intensity is saved with the attribute name Reflectance, which is a Riegl default^49^. The calibrated LiDAR intensity (Reflectance attribute) is defined by Riegl as a target property that corresponds to the fraction of incident optical power reflected by that target at a wavelength of 1,550 nm, which is corrected by range.

Georeferencing is an important step to register and analyse point clouds acquired over time and to connect the dataset to other sources of data and field references. In this regard, after point cloud rectification, a Helmert 3D transformation was performed to georeference the point cloud. The resulting Easting, Northing, and height coordinates are at the EPSG:3067 - ETRS89/ TM35FIN. The transformation involves the translation, rotation, and scaling of point cloud coordinates to align them with a reference coordinate system, which accounts for three translational parameters (along x, y, and z axes), three rotational parameters (pitch, roll, and yaw), and a scaling factor. By optimizing these transformation parameters, the point cloud is adjusted to fit accurately within the reference coordinate system, allowing for precise alignment and georeferencing. The transformation parameters were computed in a least square adjustment based on eight ground control points collected in the test area with an RTK receiver (GNSS positioning) and extracted from a georeferenced Airborne Laser Scanning (ALS)-point cloud of the area^50^. The ALS point cloud was acquired using the FGI HeliALS-TW system, which consists of a RIEGL miniVUX-1UAV scanner combined with an inertial navigation system integrated onto a helicopter platform. The FGI HeliALS-TW operated at a wavelength of 1550 nm with a flight altitude of approximately 100 meters above the ground. The ALS point cloud limited for LiPhe research area is available as additional data in LiPheStream^44^.

Point cloud data resampling was not applied during the generation of LiPheStream^44^. The individual tree point clouds are provided at full spatial resolution. However, users can resample the data as needed, particularly for standardizing and optimizing data processing. Voxelization-based resampling can be performed using LiPheKit^51^. LiPheKit’s resampling process begins by voxelizing the point cloud based on a user-defined cubic voxel size. A maximum cubic voxel size of 5 cm is recommended to achieve results comparable to those obtained with full resolution. A neighborhood search (using the KD-tree method) is then conducted within each voxel, preserving the point closest to the centroid of all points in the voxel. The average properties (e.g., intensity) of the points are calculated and assigned to the retained point. During this process, noise related to instrumentation and atmospheric influences is filtered out using Reflectance and Deviation thresholds. As additional data for user-friendly manipulation and scan area visualization, we have provided two full scene point clouds resampled using the LiPheKit voxelization-based method, with a 5 cm cubic voxel resolution.

Finally, coarse-to-fine point cloud segmentation was performed to segment individual trees from the full point cloud scene. In the first step, trees were identified by an automatic stem detection process that resulted in a stem map of a buffer area of 200 m from the scanner^45^. We consider a target-to-scanner distance of 200 meters as a reliable limit for point cloud tree detection, due to the scanner’s long range, high resolution, and its ability to capture up to 8 returns. The RIEGL VZ-2000i’s data acquisition capabilities, combined with the oblique view from above the tree line, ensure that stems remain visible within the 200-meter range as long as the line of sight is not completely occluded. The stem detection method^45^ consists of two main steps: First, stem points are extracted using a local geometry analysis. For each point, a local neighborhood is defined and the main directions of the point distribution within the neighborhood are determined using primary component analysis (PCA). The point is classified as a stem point if the main distribution direction is close to vertical to the local terrain. Next, the extracted stem points are clustered using a cylinder fitting process, thus, the stem points that belong to an identical tree are clustered into one group simultaneously with the reconstruction of the 3D cylindrical stem model of the tree. This method was originally developed for TLS point clouds that have higher point densities than the LiPhe PLS. Therefore, several parameters needed to be adjusted for LiPhe point clouds to accommodate the differences in data acquisition geometry, e.g., using larger neighborhood sizes.

Individual trees were automatically identified and classified according to a field survey within the three main species in the test area: silver birch (*Betula pendula* Roth.), Scots pine (*Pinus sylvestris* L.) and Norway spruce (*Picea abies H.* Karst.). These trees were initially segmented from the full point clouds based on the stem coordinates and a cylinder buffer with a 3.5 metre radius. A subsequent refinement in segmentation was executed within the output buffer obtained from the coarse-segmentation process. The trees identified through coarse segmentation underwent a normalization process to align them with ground levels using a digital terrain model (DTM) of the study area. The DTM of the study area was generated using the ALS dataset (additional data).

The subsequent fine-segmentation step utilizes the layer-by-layer algorithm^52^ to detect and segment individual trees within the cylindrical buffer. In the tree detection phase, the input point cloud is first divided into horizontal layers. The points are then projected onto the *xy*-plane after which the DBSCAN (Density-Based Spatial Clustering of Applications with Noise) clustering algorithm^53^ is applied to each layer separately. Subsequently, tree locations are identified by fitting vertical lines on the resulting clusters, using local maxima of the *z*-coordinates as auxiliary information. For each detected tree, the algorithm outputs a set of corresponding points, which are provided as initial training data for the segmentation step. Finally, all the points in the cylindrical buffer are iteratively classified to individual trees layer by layer using a Fuzzy k-Nearest Neighbours (FkNN) classifier^54^. Since the original layer-by-layer algorithm was designed for ALS point clouds, the algorithm parameters were specifically optimized for the LiPhe data using grid search.

As final result of the LiPhe pipeline, georeferenced individual tree point clouds are obtained. Each point cloud contains georeferenced 3D coordinates, the point return number (scalar from 1 to 15), number of returns (scalar from 1 to 15), intensity as amplitude, scan angles (theta and phi in degrees), reflectance (dB), return pulse deviation (measure of pulse shape distortion), and the 3D range from the scanner (m).

### Data selection

#### Dataset items

A total of 458 individual trees per full scene point cloud (*laz*) were selected as the final dataset for LiPheStream^44^ (Table 1). The automatic stem detection process identified a total of 633 tree stems in the test area. During the fine-segmentation process, trees with low resolution, that are absent, or that do not match the reference stem map were excluded from the dataset. In a second step, visual inspection was also performed, excluding trees at the edge of the research area (incomplete canopy) or with only the stem visible. Data selection was focused specifically on addressing the limitations of the data acquisition geometry. The single-view perspective of LiPhe data acquisition results in inherent occlusions that affect the completeness of the tree data, particularly when compared to multi-TLS scan surveys. Additionally, forest growth during spring and leaf-on periods can cause increased occlusions from underground vegetation and neighboring trees, which were not considered as factors for excluding trees from the dataset. Out of all the selected individual trees, 298 trees are Scots pine, 77 trees are Norway spruces, 79 trees are silver birch, and 4 are dead trees of an unidentified species. Since the monitored forest is dominated by Scots pine, this species proportion (3/1/1) is consistent with the distribution of species in the research area. Additional reference information of the selected trees includes the respective location, the species, and the height at the beginning of the time series. We provide as additional data in LiPheStream^44^: (1) two LiPhe point clouds (*laz*) of the whole scan area in April 2020 (*leaf-off*) and July 2020 (*leaf-on*) at two different resolutions - full resolution and 5 cm resampled resolution ; (2) a 20 cm resolution DTM (*laz*) of the research area; (3) an ALS point cloud acquired by FGI HeliALS-TW of the LiPhe research area; and (4) a vector file (*gpkg*) containing the footprint of the research area covered by LiPhe. DTM, ALS point cloud and the vector file cover the research area within a distance of 200 metres from the LiPhe laser scanner system.

**Table 1:** The number and distribution of individual trees between different tree species in the dataset, according to the data quality.

Segmentation Quality	Pine	Spruce	Birch	Other	Sum
Level 4	57	14	16	1	88
Level 3	52	16	15	2	85
Level 2	55	10	8	0	73
Level 1	59	4	4	0	67
Level 0	75	33	36	1	145
Sum	298	77	79	4	458

Class ’Other’ includes other than the three main tree species in the scan area and dead trees with undetermined species.

#### Attribute values

As metadata, each individual tree monitored in the time series come along with (1) a 3D georeferenced location (E, N, h) at ETRS89/ TM35FIN; (2) a species index, ranging from 1 to 4, meaning Scots pine, Norway spruce, silver birch and unidentified, respectively; (3) point cloud data quality (see Technical Validation section); (4) distance from the LiPhe laser scanner position to the tree crown top and the stem at ground level; (5) estimated point density per tree per square meter; and (6) the tree height at the first time point in the time series (April 6th, 2020). The tree height was calculated as the point height difference between their maximum and minimum elevations. The minimum height was estimated by identifying the nearest point from a 20-cm DTM to the tree location, approximating the ground level at its base, while the maximum height was determined as the 99.95th percentile of the H coordinates, representing the highest point(s) of the tree. The accuracy of the estimated tree height presented in the metadata is directly related to the segmentation data quality described in the Technical Validation section.

#### Geographic extent

LiPhe data collection is conducted at plot level. The forest scene is defined by a scanning window with a field of view measuring 87° vertically and 151° horizontally, comprising 19 ha. The dataset was generated focusing on those trees with higher visibility and point cloud density that are within a distance of 200 m from the scanner, comprising approximately 6 ha, with a stem density of around 625 stems per ha. Despite the small geographic extent of the dataset, it represents a sample of the predominant tree species found in Finland, namely Scots pine (*Pinus sylvestris*), Norway spruce (*Picea abies*), and silver birch (*Betula pendula Roth*.). These species are widely distributed across various forested regions of Finland and hold significant ecological and economic importance within Boreal forest ecosystems.

#### Temporal extent

**Fig. 3:**
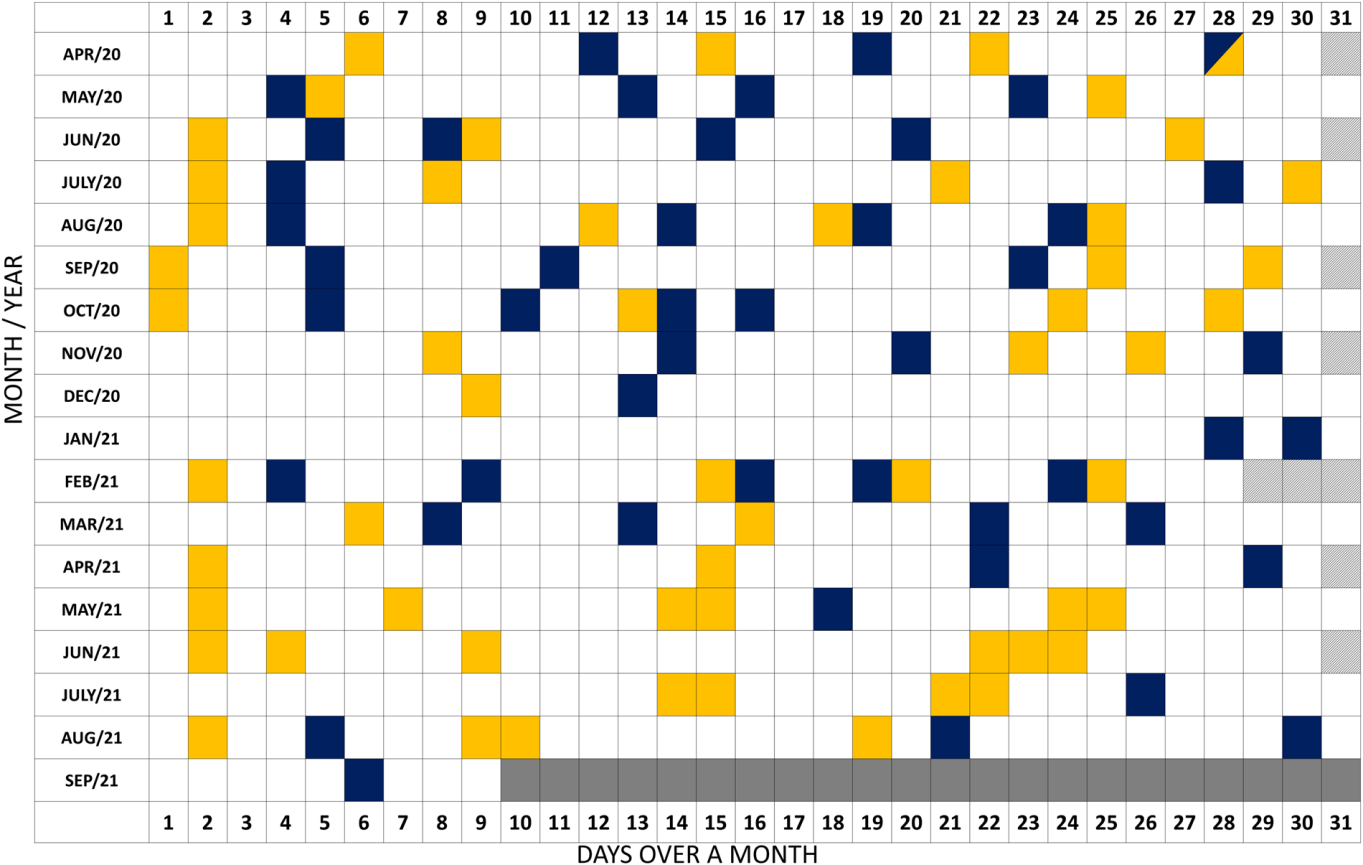
Visualization of LiPheStream temporal resolution. Date boxes highlighted in yellow and blue indicate the dataset measures, with yellow denoting daytime scans and blue nighttime scans. White date boxes represents scans that were collected by LiPhe but not selected. Grey date boxes represent periods when LiPhe was unable to collect data due to maintenance requirements.

A total of 103-point clouds were selected from the LiPhe operational period from April 2020 to September 2021. The two scans per week data set is composed of morning (after sunrise) and night (after sunset) scans with favourable weather conditions, which were set to be a wind-speed smaller than 3m/s, with no precipitation, no snow, and relative humidity less than 90 percent. During the winter period in the study site (between the middle of December 2020 and the end of February 2021), weather conditions were more challenging and only 6 scans per day were collected with the LiPhe. The winter period data consists of 12 time points in total that require additional analysis steps due to changes in tree architecture caused by snow accumulation and the elevated noise levels due to the atmospheric conditions. Local weather information used in data selection was provided by the Station for Measuring Ecosystem-Atmosphere Relations II (SMEAR II) located in the same research area^55^ and can be accessed in the SMEAR II repository (https://smear.avaa.csc.fi/). Figure 3 presents a visualization of the dates available in the LiPheStream dataset. The selected scans are colourised to indicate whether they were collected during daylight (yellow) or night periods (blue), based on Finnish sunrise and sunset times throughout the year. Most of the daylight scans were selected between 10 AM and 12 PM, while the night scans were chosen between 12 AM and 02 AM. It is worth noting that Finland experiences significant variations in daylight length, resulting in more daylight scans during the summer months and oppositely during the winter period.

## Data Records

LiPheStream^44^ includes the single tree time series and additional support data. Due to its size and bearing the potential user’s interests in mind, the dataset has been split into single tree time series in the *zip* archives format. The time series is organized by tree species and the most prominent data quality class of the dataset. The dataset folders are ordered first per species (scots_pine, silver_birch, norway_spruce and unidentified) and second by quality class of the majority of time points per tree (ranging from 0 to 4 (best)). The filenames (YYMMDD_HHMMSS_treeID_Quality.laz) indicates both the date and time (YYMMDD_HHMMSS) of the scan acquisition, the point cloud quality and the tree ID. Tree attributes and location estimated from the first point cloud in the time series on April 06, 2020 are available as metadata. The full single tree time series dataset with 1,25 Terabytes (TB) of point cloud data in *laz* format is publicly available at the Finnish Fairdata service^44^. In addition, to provide a better overview of the research area and tree neighborhoods, we have shared in LiPheStream the full scan area point clouds for LiPhe at two time points (April 6, 2020, “leaf-off” and July 15, 2020, “leaf-on”) in two resolutions (full resolution and 5 cm voxel resampled). We have also included an ALS point cloud covering the LiPhe research area within a 200-meter radius from LiPhe. With this dataset we also provide LiPheKit sample data: A polygon in *geopackage* format covering the whole area of LiPheStream tree locations, a digital terrain model for the LiPhe area and a low resolution small area scan from April 06, 2020 for code testing. The Fairdata service (https://www.fairdata.fi/en/) provides the possibility to download *zip* archives of the full dataset, individual directories and single tree time series via the web interface. To enable informed single tree dataset downloads, a *json* catalogue fulfilling the minimum requirements of the pytreedb schema (https://pytreedb.geog.uni-heidelberg.de/) is provided with the dataset. The catalogue can be used to find a particular tree time series; example usage of the catalogue is presented as a part of the LiPheKit.

## Technical Validation

ISO 19157^56^ defines the principles for describing geographical data quality with six data quality elements: positional accuracy (relative and absolute), logical consistency, completeness, temporal quality, thematic accuracy and usability. In this work, we consider that only the quality elements, positional accuracy, completeness, temporal quality, and usability are applicable for the current individual tree dataset validation.

### Positional accuracy

Positional accuracy refers to the precision of feature locations within a spatial reference system, which can be expressed by two data quality sub-elements absolute or relative positional accuracy. Absolute or external positional accuracy is defined as the proximity of reported coordinate values to those acknowledged as true. In this study, absolute positional accuracy is directly linked to the precision of the point cloud georeferencing process. We conducted a comparative analysis by comparing the georeferenced positions of tree stems detected in the point cloud against those determined through field surveys, considered as the reference. An average planimetric distance of 0.48 m was estimated between the stems detected position and the reference. We estimated the Root Mean Square Error (RMSE) of the disparities between the estimated and independently surveyed tree stem coordinates, achieving a planimetric absolute positional accuracy of 0.417 cm and 0.328 cm in the East (E) and North (N) coordinates, respectively. It is important to emphasize that accurate field surveys referenced under a canopy pose a significant challenge. Since the GNSS signal tends to be weak and RTK positioning can be affected by multipath signal return, we additionally compared the georeferenced LiPhe point cloud with an ALS-derived point cloud collected within the same hour and plot area. An average point-to-point distance of less than 15 cm was found, with 71.3 percent of all the points located in the scan area being within this threshold^50^. Discrepancies larger than 15 cm may be due to variations in perspectives between PLS and ALS, particularly considering that some tree canopies were not fully visible to the LiPhe scanner. The dynamics of the scene are also a challenge when registering or quantifying uncertainties^25^. Therefore, to integrate this dataset with other georeferenced external datasets, we recommend fine-registration methods such as the iterative closest point (ICP) algorithm^57^. Relative or internal accuracy involves the proximity of the relative positions of features in a dataset to their corresponding relative positions acknowledged as accurate or true. By the quality of the registration process, we evaluate the relative positional accuracy of the individual tree point clouds. When comparing two LiPhe point clouds acquired in the same day, 98 percent of point-to-point distances were smaller than 15 cm, with an average point-to-point distance smaller than 0.01 metres. This indicates high consistency between the point clouds in the time series. A relative accuracy (RMSE) of 5 cm was estimated for two point clouds collected within the same day.

### Completeness

**Fig. 4:**
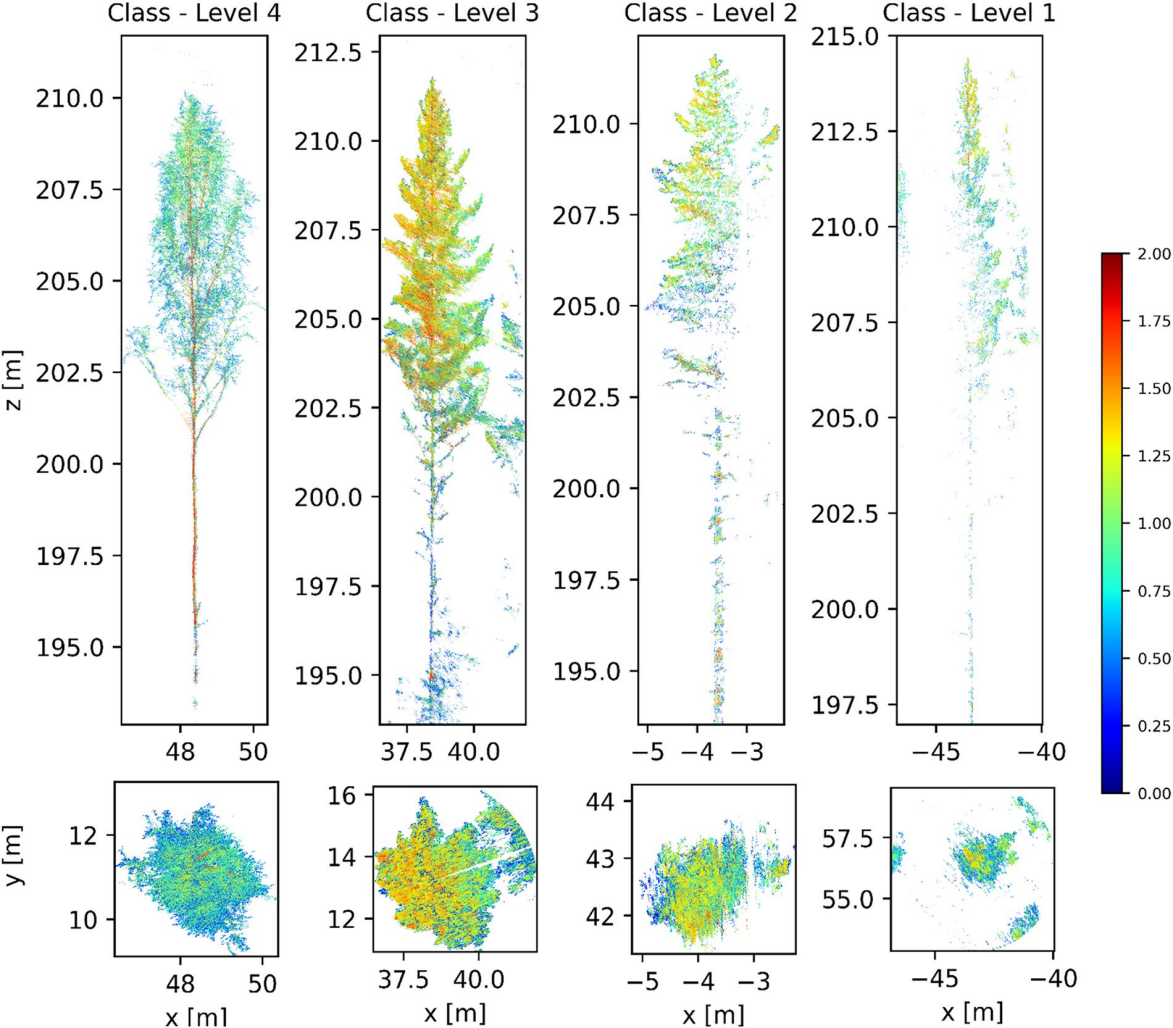
Examples of different trees with different segmentation accuracy in frontal (x,z) at upper panel and top-down view (x,y) at bottom panel. Fully segmented with high accuracy, no commission or omission (Class level 4); segmented with commission but no omission (Class level 3); segmented with no commission but with omission (Class level 2); and segmented with both commission and omission (Class level 1).Trees are colorized by normalized LiDAR-Reflectance values ranging from 0 to 2.

Completeness analysis consists of identifying the excess (commission) and the absence (omission) of data (features, attributes and relationships) in a dataset compared with its specification. Here, we consider as commission the individual tree point cloud that has extra points that are not part of the tree structure, which is directly related to the quality of the segmentation process (noise points). As omission, we consider individual tree point clouds that have part of its structure occluded due to the data acquisition geometry or that were digitized with a low resolution, for instance, individual trees that have part of the canopy or stem missing. In this regard, the final 458 individual trees were manually labelled by an expert visual inspection, according to data commission or omission. It is important to highlight that in our dataset, omission and commission can occur simultaneously, when points that are not part of tree structure were digitized but part of the tree structure was lost. In this regard, we classify all of the tree structure according to four completeness levels. Individual point clouds with no commission (satisfactory fine-segmentation results) and no omission (stem and canopy visible) are flagged as level 4. Individual point clouds with commission (unsatisfactory fine-segmentation results) and no omission (stem and canopy visible) are flagged as level 3. Level 2 is assigned for Individual point clouds with no commission (satisfactory fine-segmentation results) but with omission (stem and/or canopy are invisible). Finally, individual point clouds with commission (unsatisfactory fine-segmentation results) and with omission (stem and/or canopy are invisible) are flagged as level 1. It is important to highlight that different levels of commission and omission are presented inside this category. In addition, level 0 are single tree point clouds for which the fine segmentation step failed. Figure 4 shows examples of trees classified as completeness levels 4 to 1.

### Temporal quality

The quality of a temporal resolution can be understood as the closeness number of time measurements in relation to the total number of time points accepted as a reference for the dataset purpose. The dataset goal was to monitor weekly forest events and changes from two growth seasons. A total of 103 time points were extracted, providing more than one scan per week from April 2020 to September 2021. For comparison, when considering publicly available optical Earth Observation data acquired by Sentinel-2, about 25 nearly cloud-free observations could be obtained of the same area and timeframe. Figure 3 shows the distribution of the time points for the laser scanning data. A nonuniform distribution can be observed due to the selection of timepoints according to weather conditions. The most challenging months for data acquisition without climatic interference were November, December and January, due to precipitation and potential snow events, as well as, both Julies 2020 and 2021, due to high wind speeds (>3 m/s) recorded in the research area.

### Usability

The presented dataset is expected to be of high value to the scientific community. The dataset provides high spatial and temporal resolution obtained with a non-destructive forest monitoring technique. The LiPheStream dataset^44^ offers opportunities for both forest analyses and developing point cloud data processing methodologies for forest applications. From an ecological point of view, the dataset allows future investigations into seasonal forest dynamics. These include inter- and intra-species variability in tree growth, changes in aboveground biomass, and estimating EBVs like phenological timing (e.g. spring sprouting and flowering) in the stand trees. For instance, a similar LiPhe dataset has already been used to investigate the relationship between the height and diameter growth dynamics of the three dominant tree species in the LiPhe scan area. The study showed a quantifiable link between the height growth derived from the LiPhe time series, dendrometer measurements, and local environmental variables^43^. Other recent studies utilizing LiPhe data demonstrate the potential to investigate differences in silver birch (*Betula pendula,* Roth.) growth dynamics^42^, and phenology timing^10^, showing that intra-species differences can be observed even in a geographically limited area under similar weather conditions. When compared with results from allometric models, consistent results in biomass estimation using LiPhe data and quantitative structure models have been reported^58^. LiPheStream^44^ dataset also enables the discovery of new possibilities of LiDAR time series in forest and forest ecology applications. Recent studies using the LiPhe dataset have demonstrated how a tree species can be classified using a dense single wavelength LiDAR time series alone^41^. The dataset could also potentially be used to geometrically reconstruct the occluded part of the Hyytiälä forest^59^. However, the quality of tree attributes extracted from the LiPheStream dataset must be carefully assessed by dataset users. The tree point cloud quality parameters (e.g., spatial accuracy and completeness) need to be considered case by case, regarding the intended forest application. Data selection may be necessary. The quality of tree point cloud time series is susceptible to various sources of error, such as instrumental noise, environmental conditions like snow accumulation, data processing performance, and especially occlusions. Despite the penetration capability of LiDAR, the single-view perspective of the LiPhe data acquisition does not provide full coverage of the tree’s backside, particularly when compared to multi-TLS scan surveys. Additionally, forest growth during spring and leaf-on periods reduces laser beam penetration through the canopy and increases occlusion due to understory vegetation and neighboring trees growth.

From a LiDAR data processing point of view, the mentioned forest dynamics investigations are more valuable when performed at individual tree level. This level of detail requires an automated workflow from the full scene to individual tree point clouds over time. Therefore, the LiPheStream dataset^44^ can also be used as a benchmark for LiDAR data processing methods and computational performance optimizations. For instance, tree parameter extraction from point clouds acquired over time requires complex data processing methodologies and high computational resources, which is still under development by the LiDAR community. Recently, the example codes provided with the LiPheKit^51^ were integrated into workflow management tools for efficiently processing large datasets^60^, demonstrating the usability of LiPheStream dataset and the LiPheKit for assessing computational performance optimizations. Workflow management tools like Snakemake^61^ provide a wide variety of features like implicit parallelization, re-entrance, error recovery strategies, and output validation for more scalable, manageable and efficient processing.

## Usage Notes

For convenience, the dataset has been arranged by species names and quality class for publication. Each single tree time series is provided in a single *zip* file containing single tree - single time point *laz* files. The most accurate metadata and complete tree data can be obtained by only utilizing the data of quality class 4. The quality class is determined by the quality class of the majority of time points in the time series. This means, that even if a tree is located in the quality class 4 folder, some time points may include lower quality data. The provided single tree - single time point *laz* files can be further processed or visualized with any software product that can read *laz* files, for example CloudCompare (https://www.danielgm.net/cc/), lastools (https://lastools.github.io/), Python’s laspy (https://laspy.readthedocs.io/en/latest/), R’s lidR (https://r-lidar.github.io/lidRbook/). Please note that not all of these tools may be able to read all extrabytes stored in the LiPheStream *laz* files.

## Data Availability

Alongside this dataset, we also provide the LiPheKit^51^, including the code to produce a dataset like LiPheStream^44^ from full scan point cloud data. The code for the full processing chain is publicly available in the gitlab repository^51^. The workflow code covers the workflow starting from the full scan *laz* file to the fine segmented single tree point clouds and extraction of single tree metadata (Fig. 2). The code is licensed under an MIT license and can be reused with other point cloud datasets by adapting the included configuration files. All required external tools and their version numbers used in producing the current dataset are provided in the *requirements.txt* file in the repository (https://gitlab.com/fgi_nls/public/liphekit/-/blob/main/requirements.txt). The LiPheKit also includes the code to produce the single tree *geojson* catalogue files as well as examples on how to query the catalog. The LiPheStream dataset includes a separate sample data folder to test the LiPheKit functionality^44^.
